# Differential roles for cryptochromes in the mammalian retinal clock

**DOI:** 10.1096/fj.201701165RR

**Published:** 2018-03-21

**Authors:** Jovi C. Y. Wong, Nicola J. Smyllie, Gareth T. Banks, Carina A. Pothecary, Alun R. Barnard, Elizabeth S. Maywood, Aarti Jagannath, Steven Hughes, Gijsbertus T. J. van der Horst, Robert E. MacLaren, Mark W. Hankins, Michael H. Hastings, Patrick M. Nolan, Russell G. Foster, Stuart N. Peirson

**Affiliations:** * Sleep and Circadian Neuroscience Institute, University of Oxford, Oxford, United Kingdom;; † Medical Research Council (MRC) Laboratory of Molecular Biology, Cambridge, United Kingdom;; ‡ Medical Research Council (MRC) Harwell, Harwell Science and Innovation Campus, Harwell, United Kingdom;; § Nuffield Laboratory of Ophthalmology, Nuffield Department of Clinical Neurosciences, University of Oxford, Oxford, United Kingdom;; ¶ Department of Molecular Genetics, Erasmus University Medical Center, Rotterdam, The Netherlands

**Keywords:** circadian rhythms, retina, electroretinogram, pupillary light response, contrast sensitivity

## Abstract

Cryptochromes 1 and 2 (CRY1/2) are key components of the negative limb of the mammalian circadian clock. Like many peripheral tissues, *Cry1* and *-2* are expressed in the retina, where they are thought to play a role in regulating rhythmic physiology. However, studies differ in consensus as to their localization and function, and CRY1 immunostaining has not been convincingly demonstrated in the retina. Here we describe the expression and function of CRY1 and -2 in the mouse retina in both sexes. Unexpectedly, we show that CRY1 is expressed throughout all retinal layers, whereas CRY2 is restricted to the photoreceptor layer. Retinal period 2::luciferase recordings from CRY1-deficient mice show reduced clock robustness and stability, while those from CRY2-deficient mice show normal, albeit long-period, rhythms. In functional studies, we then investigated well-defined rhythms in retinal physiology. Rhythms in the photopic electroretinogram, contrast sensitivity, and pupillary light response were all severely attenuated or abolished in CRY1-deficient mice. In contrast, these physiological rhythms are largely unaffected in mice lacking CRY2, and only photopic electroretinogram rhythms are affected. Together, our data suggest that CRY1 is an essential component of the mammalian retinal clock, whereas CRY2 has a more limited role.—Wong, J. C. Y., Smyllie, N. J., Banks, G. T., Pothecary, C. A., Barnard, A. R., Maywood, E. S., Jagannath, A., Hughes, S., van der Horst, G. T. J., MacLaren, R. E., Hankins, M. W., Hastings, M. H., Nolan, P. M., Foster, R. G., Peirson, S. N. Differential roles for cryptochromes in the mammalian retinal clock.

Endogenous circadian rhythms enable organisms to anticipate and adapt to the predictable environmental changes that occur as a result of the 24-h light/dark (LD) cycle. As well as dramatic changes in activity and rest, rhythms exist in many physiologic parameters such as body temperature and hormone levels. The master circadian pacemaker is located in the suprachiasmatic nuclei (SCN) of the anterior hypothalamus (1), and it is based on an intracellular clock mechanism comprising a transcriptional–translational feedback loop (TTFL) (1). Cryptochromes (CRYs) play a key role in the TTFL, and together with their period (PER) binding partners, they form the negative limb of the feedback loop (2). In addition to the SCN, the mammalian retina also contains an autonomous circadian clock, optimizing physiology to the dramatic variations in the LD cycle (3).

The functions of CRYs in the retina have been the subject of considerable interest. Their role as photoreceptive molecules in plants and insects has led to speculation that CRYs may act as the retinal photoreceptors that detect light and transmit photic information to the SCN to entrain rhythms in physiology and behavior (4). The nature of this photopigment was subsequently resolved with the identification of the melanopsin (OPN4)-expressing intrinsically photosensitive retinal ganglion cells (ipRGCs) (5–8). The demonstration that rod, cone, and OPN4-based photoreceptors accounted for all responses to light (9) provided the conclusive evidence that CRYs did not play a role in circadian photoreception.

The retina contains all of the key elements of an independent circadian system, namely a photoreceptor, oscillator, and tissue-specific outputs. However, attempts to identify the rhythm-generating cells of the retinal circadian clock have yielded conflicting results. Dopaminergic amacrine cells have been suggested as circadian pacemaker cells, as *Per1* (10) and CRY2 (11) have been described as oscillating in these cells. Dopamine appears to act as a synchronizing signal for the retinal clock (12, 13). While dopamine may phase-shift retinal rhythms (14), loss of retinal dopamine does not affect the persistence of retinal period 2::luciferase (PER2::LUC) rhythms (12). The consensus among most studies regarding clock gene and protein expression in the retina suggests that the majority of retinal cell types express clock genes (11, 14, 15), and therefore all of these cells have the potential to act as circadian oscillators. Whether retinal cells expressing clock genes act synchronously or can be subdivided into interacting autonomous networks remains unclear.

To date, studies investigating the role of CRYs in the mammalian retina have used mice lacking both *Cry1* and *Cry2* (*Cry1^−/−^;Cry2^−/−^*). Effects on non–image-forming responses to light in double-knockout mice were taken as evidence that CRYs functioned as photopigments (16–19). However, although *Cry1^−/−^;Cry2^−/−^* double-knockout mice demonstrate negative masking, under constant conditions, these animals are arrhythmic (20). As such, altered responses in *Cry1^−/−^;Cry2^−/−^* mice may be affected by loss of circadian gating of photic input. Indeed, attenuated pupillary responses in *Cry1^−/−^;Cry2^−/−^* mice (18) were subsequently found to be due to loss of circadian rhythms, as similar phenotypes were observed in other clock mutants (21).

Given the critical role of CRYs in the SCN oscillator and the reported absence of CRY1 in the retina, we sought to define the roles of CRY1 and CRY2 in the generation of retinal circadian rhythms. To this end, we characterized the expression of these proteins in the mouse retina and then studied rhythms in molecular clock function and retinal physiology in *Cry1^−/−^* and *Cry2^−/−^* single-knockout mice.

## MATERIALS AND METHODS

### Animals

Wild-type (WT) C57BL/6J, *Cry1^−/−^*, *Cry2^−/−^* (20), and PER2::LUC (22) mice were used. *Cry1^−/−^* and *Cry2^−/−^* mice were maintained as homozygous lines. Congenic WT C57BL/6J mice were used as controls. Both male and female animals were used in experiments, and no differences were observed between sexes. Unless otherwise stated, all mice were housed under a 12:12 LD cycle with food and water *ad libitum*. All procedures were conducted in accordance with the United Kingdom Home Office Animals (Scientific Procedures) Act 1986 (PPL 70/6382, 30/2812, and 70/8090, PIL for JW I53D8E9E7) and the University of Oxford Policy on the Use of Animals in Scientific Research. All procedures were performed in a designated establishment.

### Immunohistochemistry

Retinal sections were prepared as previously described (23). Tissue was collected at relevant time points from both male and female mice. Primary antibodies were incubated for 24 to 72 h at 4°C. Primary antibodies used included rabbit polyclonal anti-CRY1 (1:200) and rabbit polyclonal anti-CRY2 (1:200) antibodies previously described (24) raised against murine peptide sequences SQEEDAQSVGPKVQRQSSN and VTEMPTQEPASKDS, respectively. In addition, other antibodies investigated include goat polyclonal anti–ultraviolet-sensitive (UVS) opsin (1:1000, Sc-14363; Santa Cruz Biotechnology, Dallas, TX, USA), rabbit polyclonal anti–arrestin C (1:1000, AB15282; MilliporeSigma, Burlington, MA, USA), chicken polyclonal anti–tyrosine hydroxylase (1:1000, ab76442; Abcam, Cambridge, United Kingdom), mouse monoclonal anti-GABA (1:2500, GB-69; MilliporeSigma), goat polyclonal anti–glycine transporter 1 (1:1000, AB1770; EMD Millipore), chicken polyclonal anti–green fluorescent protein (1:1000, GFP-1020; Aves Labs, Tigard, OR, USA), rabbit polyclonal anti-melanopsin (1:2500, UF006; Advanced Targeting Systems, San Diego, CA, USA), and chicken polyclonal anti-Per1 (1:100, PER13-A; Alpha Diagnostic International, San Antonio, TX, USA). Secondary antibodies were Alexa Fluor 488– and Alexa Fluor 568–conjugated donkey anti-rabbit, donkey anti-goat, donkey anti-mouse (Thermo Fisher Scientific, Waltham, USA), and donkey anti-chicken (Jackson ImmunoResearch Laboratories, West Grove, PA, USA) incubated for 2 h at room temperature (1:200). All antibodies were diluted in PBS with 2.5% donkey serum and 0.2% Triton X-100 (3% Triton X-100 for brain sections). Wash steps were performed using PBS with 0.05% Tween 20. Tissue sections were mounted onto glass slides with ProLong Gold antifade mounting medium containing DAPI (Life Technologies). For double-labeling experiments, both primary and secondary antibodies were incubated simultaneously. Because both CRY1 and melanopsin antibodies are raised in rabbit, colocalization of CRY1 within ipRGCs was performed using anti-CRY1 and anti–green fluorescent protein antibodies on retinal sections from OPN4.Cre.eYFP mice obtained from previous studies (25).

### Image acquisition

Images were acquired as previously described (23). An LSM 710 laser scanning confocal microscope was used together with Zen 2009 image acquisition software (Carl Zeiss, Jena, Germany). Individual color channels were collected sequentially. Laser lines (405, 488, 561, and 633 nm) were used for excitation. Fluorescence emissions at 440 to 480, 505 to 550, 580 to 625, and 650 to 700 nm were collected for blue, green, red, and far-red fluorescence, respectively. For all images, brightness and contrast enhancement was performed by ImageJ software (Image Processing and Analysis in Java; National Institutes of Health, Bethesda, MD, USA; *https://imagej.nih.gov/ij/*). For quantitative comparisons, all images were collected and processed under identical conditions.

### PER2::LUC bioluminescence recordings

Whole-retina explants were prepared from adult mice of both sexes (>30 d old) and mounted on Millicell culture inserts (EMD Millipore). Retinas were maintained in culture for 24 h before recording bioluminescence rhythms as previously described (26). Whole-retina bioluminescence emissions were measured by photon multiplier tubes (Hamamatsu Photonics, Hamamatsu, Japan) for at least 5 d. Retina were maintained at 37°C throughout. Circadian period was assessed over the course of the recording by measuring the peak-to-peak time intervals of PER2 bioluminescence.

### *In vivo* circadian retinal physiology testing

Circadian physiology testing was performed after 1 d in constant darkness at circadian time (CT) 6 ± 1 h (subjective midday) and CT18 ± 1 h (subjective midnight). Furthermore, diurnal physiology recordings were made at midday [zeitgeber time (ZT) 6 ± 1 h] and midnight (ZT18 ± 1 h) hours in the LD cycle. Sample size and sex were as follows: electroretinography, WT (*n* = 5; 3 male and 2 female), *Cry1^−/−^* (*n* = 7; 4 male and 3 female) and *Cry2^−/−^* (*n* = 7; 4 male and 3 female). Contrast sensitivity, WT (*n* = 7, all male), *Cry1^−/−^* (*n* = 10, all male), *Cry2^−/−^* (*n* = 10, all male). Pupillometry, WT (*n* = 5; 3 male and 2 female), *Cry1^−/−^* (*n* = 7; 4 male and 3 female) and *Cry2^−/−^* (*n* = 7; 4 male and 3 female). Unless otherwise stated, each mouse was tested at all 4 testing times (ZT6, ZT18, CT6, CT18) in a random order. Finally, to reduce light adaptation effects on dark-adapted mice, testing periods for individual mice were restricted to less than 20 min from first exposure to light.

### Electroretinography

Before electroretinography, general anesthesia was induced in mice by a single intraperitoneal injection of medetomidine hydrochloride (1 mg/kg body weight; Dormitor, Pfizer, New York, NY, USA) and ketamine (60 mg/kg body weight; Ketaset, Fort Dodge Pharmaceuticals, Fort Dodge, IA, USA), and pupils were fully dilated with 1% tropicamide and 2.5% phenylephrine hydrochloride eye drops (Bausch + Lomb, Rochester, NY, USA). Electroretinography recordings were performed as previously described (Espion E2; Diagnosys, Lowell, MA, USA) (27). Photopic responses were recorded after overnight dark adaptation (except for the case of ZT6 recordings, which were undertaken at ZT6 after 1 to 2 h of dark adaptation). White flash stimuli (4 ms) were superimposed on a light-adapting 30 cd/m^2^ white background with 20 responses averaged using an interstimulus interval of 1 s. Averaged flash stimuli recordings were taken every 2.5 ± 1 min from the first exposure to the white background (0 s) for 25 min in total. In a single case (*Cry1^−/−^* mouse electroretinogram (ERG) recorded at CT18 after 17.5 min light adaptation), a single data point was unobtainable due to technical problems. The b-wave amplitudes were quantified with specialized software (Espion; Diagnosys). At the end of the procedure, general anesthesia was reversed by intraperitoneal injection of atipamezole hydrochloride (5 mg/kg body weight; atipamezole; Pfizer).

### Optomotor responses

Optokinetic head tracking (an optomotor response test) was used to measure contrast sensitivity and visual acuity using the OptoMotry system (CerebralMechanics, Lethbridge, AB, Canada) as previously described (28). The test arena inside the OptoMotry system composed of a sine-wave grating projected by 4 interfacing LCD monitors rotating in one direction. The direction of rotation was randomized for each mouse. Mice were placed on a raised platform inside the OptoMotry system, and head tracking responses were recorded. Visual acuity was recorded first using a staircase method for a black-and-white sinusoidal grating at 100% contrast to determine spatial frequency threshold for visual acuity. Visual acuity was recorded as the spatial frequency where head tracking stopped. For contrast sensitivity testing, a black-and-white sinusoidal grating at 100% contrast was reduced until head tracking stopped at the contrast threshold. Six standard spatial frequencies (0.031, 0.064, 0.092, 0.103, 0.192, and 0.272 cycles/degree) were tested. Contrast sensitivity was taken as the reciprocal of the contrast threshold. All measurements were made under photopic conditions. At 100% contrast, 0.3 cycles/degree, 12 degrees/s, light intensity as measured at the platform was 50 lx, 50% contrast was 40 lx, and 10% contrast was 32 lx. The order of spatial frequency tested was randomized for each mouse.

### Pupillometry

Pupil light responses were measured as previously described (29, 30). For recordings under diurnal conditions, mice were adapted to the dark for 1 to 2 h before testing. A xenon arc lamp (150 W solar simulator; Lot Oriel, Leatherhead, United Kingdom) with a 480 nm monochromatic filter (10 nm half-bandwidth; Andover, Salem, NH, USA) was used to produce a light intensity of 14.6 log quanta/cm^2^/s (173 μW/cm^2^/s). Irradiance measurements were made using a radiometrically calibrated spectrophotometer (Ocean Optics, Oxford, United Kingdom). Light stimuli (10 s) were transmitted to the eye *via* a liquid light guide as an irradiant light stimulus using a 50.8 mm integrating sphere (Prolite Technology, Cheltenham, United Kingdom) and was controlled by a shutter positioned in the light path [LSZ160 shutter; Lot Oriel; custom software supplied by Brian Reece Scientific (BRSL), Newbury, United Kingdom]. Images of consensual pupil responses were collected with a Prosilica NIR-sensitive CCD video camera (BRSL) at a rate of 10 frames/s under infrared LED illumination (850, 10 nm half-bandwidth). During pupil measurements, unanesthetized animals were temporarily restrained using normal husbandry techniques for the duration of the recording (29 s, including baseline, stimulation, and recovery phases). Each animal was handled and pupil light responses recorded at least twice several days before the test in order to minimize any artifacts due to handling and procedure-related stress. All images were analyzed by ImageJ software.

### Statistical analysis

Data are presented ± sem. Statistical analysis was performed by paired 2-way ANOVA with *post hoc* testing or paired/unpaired 2-tailed Student’s *t* test by GraphPad Prism 6 (GraphPad Software, La Jolla, CA, USA).

## RESULTS

### CRY1 is expressed in photoreceptors and amacrine cells

**Figure 1:**
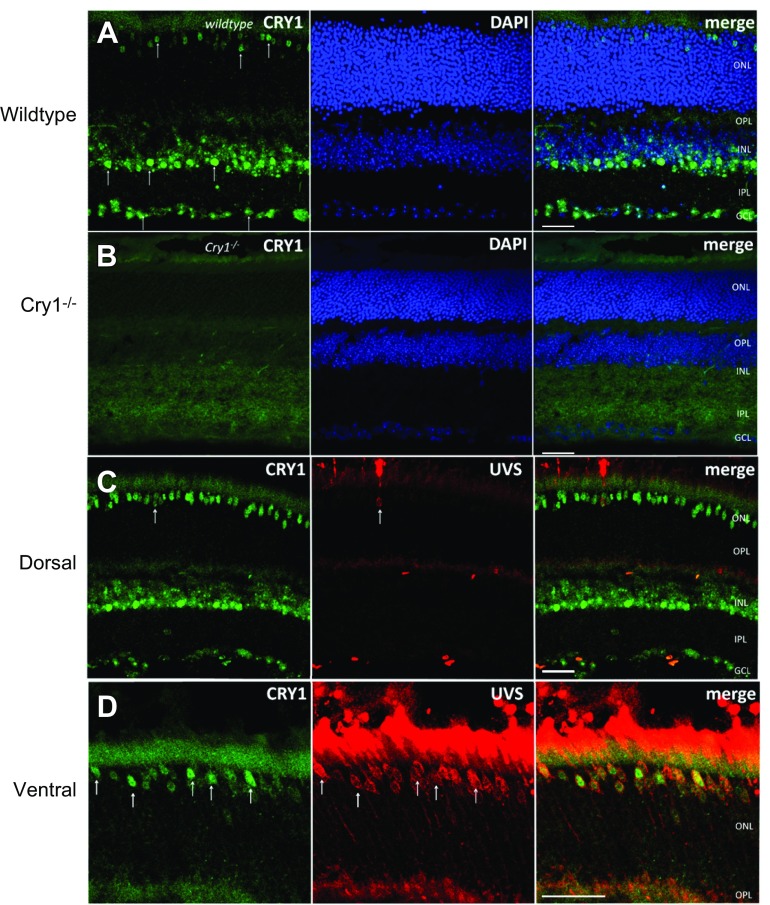
CRY1 expression in mouse retina. *A*) CRY1 (green) is expressed in cones and amacrine cells, with some expression in ganglion cell layer. DAPI staining is shown in blue. *B*) CRY1 (green) is not detected in *Cry1^−/−^* retina. *C*, *D*) CRY1 (green) colocalizes with UVS opsin (red) in both dorsal (*C*) and ventral (*D*) regions of retina. Scale bar, 20 μm.

Initial localization of CRY1 and -2 in the mouse retina was conducted using commercially available antisera (Alpha Diagnostic International) as previously described (11). Labeling with this anti-CRY1 antisera did not result in any significant immunoreactivity in the mouse retina, whereas labeling with anti-CRY2 resulted in a pattern of immunoreactivity similar to that previously described. However, this antisera was found to produce a comparable signal after incubation with retina tissue from *Cry2^−/−^* mice, suggesting a lack of specificity (Supplemental Fig. S1*A*, *B*). As such, alternative polyclonal antisera against mouse CRY1 and -2 (24, 31) were used to determine the localization of CRY1 and -2 protein in mouse retinal sections (ZT2 to -10) (**Fig. 1** and Supplemental Fig. S1*C*, *D*). The specificity of CRY1 and -2 antisera was confirmed by staining of HEK293T cells transiently transfected with plasmids encoding either *Cry1* or *Cry2* tagged with human influenza hemagglutinin (Supplemental Fig. S2). Anti-hemagglutinin staining was used as a positive control to confirm cell transfection in the absence of specific anti-CRY labeling.

CRY1 labeling was restricted to retinal cells in the outer nuclear layer, inner nuclear layer, and ganglion cell layer, consistent with expression of CRY1 predominantly within cone photoreceptors and amacrine cells (Fig. 1*A*). Costaining with DAPI showed that both cytoplasmic and nuclear punctate staining was observed. *Cry1^−/−^* retinal tissue was used as a control and showed no specific CRY1 signal (Fig. 1*B*). CRY2 localization was again inconclusive, and other than some elevated signal in the photoreceptor layer (Fig. 1*C*), similar results were obtained in WT and *Cry2^−/−^* retinal tissue (Supplemental Fig. S1*D*). Furthermore, retinal tissue was obtained at several time points (ZT2, -6, -10, -14, -18, and -22) and stained for CRY1 and -2. This time course of data revealed no significant changes in expression levels of CRY1 or -2 over the sampled times that were detectable by immunohistochemistry methods (data not shown).

### CRY1 is expressed in all cones of mouse retina

**Figure 2:**
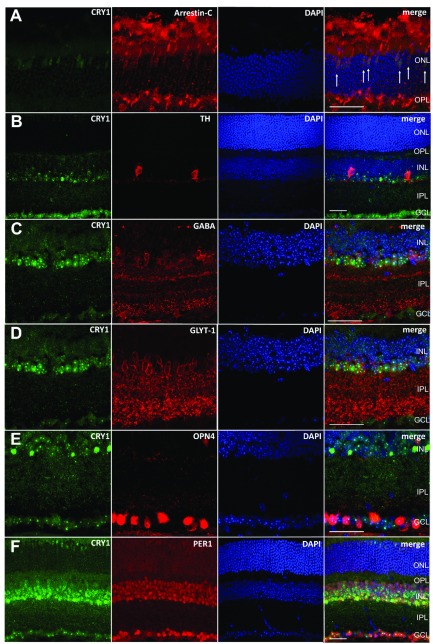
CRY1 fully coexpresses with all cones and partially coexpresses with several retinal markers. DAPI staining is shown in blue, and merge shows all channels merged together. Scale bar, 20 μm. *A*) CRY1 (green) coexpresses fully with cone arrestin (red). *B*) CRY1 (green) partially coexpresses with dopaminergic amacrine cells, marked by tyrosine hydroxylase expression (red). *C*) CRY1 (green) partially coexpresses with GABAergic amacrine cells, marked by GABA expression (red). *D*) CRY1 (green) partially coexpresses with glycinergic amacrine cells, marked by glycine transporter 1 (GLT-1) expression (red). *E*) CRY1 (green) partially coexpresses with ipRGCs, marked by eYFP expression (red, in retina tissue from Opn4.Cre.eYFP mice). *F*) CRY1 (green) partially coexpresses with clock protein PER1 (red). Note that GABA and GLYT-1 colocalization with CRY1 was conducted on the same sections.

To confirm the expression of CRY1 in cone photoreceptors, CRY1 was coimmunolabeled with UVS opsin, which labels both S cones and the majority of M cones within the mouse retina (23, 32). CRY1 expression was detected within all UVS opsin–positive cones located in the dorsal and ventral retina, and was also observed in UVS opsin negative M cones of the dorsal retina (Fig. 1*C*, *D*) (23). These data indicate that CRY1 is expressed in all cones of the mouse retina, including M cones and S cones. To confirm this, cone arrestin (a marker of all cones) was also studied showing colocalization with CRY1 (**Fig. 2*A***). Furthermore, to confirm the expression of CRY1 in amacrine cells (and to a lesser extent retinal ganglion cells), colocalization studies were performed with a set of known retinal cell markers. CRY1 was found to partially colocalize with tyrosine hydroxylase (a marker of dopaminergic amacrine cells; Fig. 2*B*), GABA (a marker of GABAergic amacrine cells, Fig. 2*C*), glycine transporter 1 (a marker of glycinergic amacrine cells, Fig. 2*D*), and melanopsin-expressing ipRGCs [labeled using enhanced yellow fluorescent protein (eYFP)] in retina sections from *Opn4^+/−^*;Cre^+/−^;eYFP mice Fig. 2*E*). Moreover, CRY1 was also found to partially colocalize with the clock protein PER1 (Fig. 2*F*).

### *Cry1^−/−^* mice show low-amplitude, unstable retinal circadian rhythms

**Figure 3:**
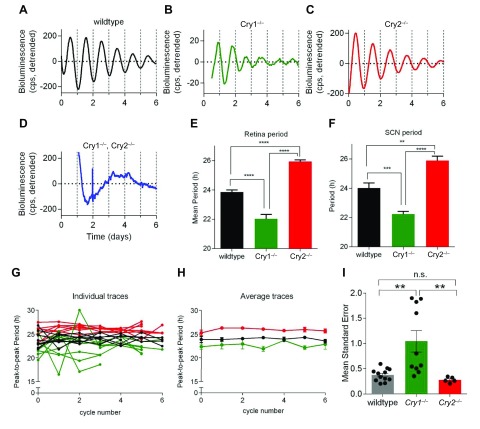
*Cry1^−/−^* mice have unstable retinal circadian rhythms, while both *Cry2^−/−^* and WT mice have normal retinal rhythmicity. *A*–*D*) Representative PER2::LUC bioluminescence traces from organotypic retina whole mounts taken from WT (black, *A*), *Cry1^−/−^* (green; *B*), *Cry2^−/−^* (red; *C*), and *Cry1^−/−^;Cry2^−/−^* (blue; *D*) animals. *E*, *F*) Group data (means ± sem) for circadian period of retina (*E*) and SCN organotypic slices (*F*). Absence of CRY1 significantly decreased period, and absence of CRY2 significantly increased period compared to WT for both tissue types (*n* > 4/group). One-way ANOVA with Tukey’s multiple comparisons test. **P < 0.005, ****P < 0.0001. *G*) Peak-to-peak period measurements of PER2::LUC bioluminescence rhythms in individual retina explants taken from WT (black), *Cry1^−/−^* (green), and *Cry2^−/−^* (red) animals. Fluctuations in circadian period were monitored across recording time frame, revealing particularly striking period fluctuations in *Cry1^−/−^* retinas (green). *H*) Group data (*n* > 4/group; means ± sem) showing peak-to-peak period of retina explants using data shown in *A*. *Cry1^−/−^* retina (green) shows larger error bars than WT retina (black) or *Cry2^−/−^* retina (red). *I*) sem of peak-to-peak period measures calculated for each recording (black dots), as proxy of stability of circadian period. Group data (*n* > 4/group; means ± sem) indicated that *Cry1^−/−^* retina rhythms were significantly less stable (had higher sem) than WT and *Cry2^−/−^* retinas. **P < 0.005 (1-way ANOVA with Tukey’s comparisons test).

PER2::LUC reporter mice (22) were crossed with either *Cry1^−/−^* or *Cry2^−/−^* mice in order to determine the effect of CRY loss on retinal and SCN circadian rhythms. WT, *Cry1^−/−^*, and *Cry2^−/−^* retinal explants all demonstrated circadian rhythms over multiple cycles, whereas *Cry1^−/−^*; *Cry2^−/−^* mice were arrhythmic (individual traces shown in **Fig. 3*A–D***). The period of circadian rhythms in both retinal and SCN PER2::LUC bioluminescence significantly shortened in *Cry1^−/−^* mice and significantly lengthened in *Cry2^−/−^* mice compared to WT mice. WT mice (*n* = 12) exhibited a retinal circadian rhythm with a period of 23.9 ± 0.14 h, whereas *Cry1^−/−^* mice (*n* = 11) had a retinal rhythm period of 21.9 ± 0.50 h and *Cry2^−/−^* mice (*n* = 5) a period of 25.9 ± 0.11 h (Fig. 3*E*). This was consistent with measurements in organotypic SCN slice preparations. WT mice (*n* = 7) demonstrated an SCN circadian rhythm with a period of 24.0 ± 0.35 h, whereas *Cry1^−/−^* mice (*n* = 7) had a retinal rhythm period of 22.1 ± 0.28 h and *Cry2^−/−^* mice (*n* = 5) a period of 25.9 ± 0.30 h (Fig. 3*F*).

When comparing the peak-to-peak period measurements of PER2::LUC bioluminescence rhythms in individual retina explants, we found that *Cry1^−/−^* mice have unstable retinal circadian rhythms compared to either *Cry2^−/−^* or WT animals. Individual retina explant data (Fig. 3*G*) and group-averaged data (Fig. 3*H*) show that *Cry1^−/−^* mice exhibit greater fluctuations in period throughout the duration of the recordings compared to either WT or *Cry2^−/−^* mice. Further analysis of the sem, as a proxy measure for circadian rhythm period robustness and stability, then revealed that *Cry1^−/−^* retinas have a significantly greater variability—that is, a greater instability in circadian period—compared to WT or *Cry2^−/−^* retinas (Fig. 3*I*). The means ± sem for retina peak-to-peak period measurements in WT mice were 0.377 ± 0.036. In *Cry1^−/−^* mice, the mean standard error was 1.04 ± 0.21, while in In *Cry2^−/−^* mice, the mean standard error was 0.281 ± 0.025. One-way ANOVA revealed a significant difference between retina sems (*F*_(2, 24)_ = 8.77, *P* = 0.0014), and Tukey’s *post hoc* multiple comparisons tests revealed a significant difference in sem between WT and *Cry1^−/−^*, as well as between *Cry1^−/−^* and *Cry2^−/−^*, but not between WT or *Cry2^−/−^*.

### Rhythms in photopic ERG b-wave amplitude are attenuated in *Cry1^−/−^* and *Cry2^−/−^* mice

Given the observed differences in retinal expression patterns and effects on retinal clock amplitude and stability, we proceeded to investigate whether CRY1 and -2 may have differential effects on the regulation of rhythmic retinal physiology. The photopic (light adapted) ERG b-wave amplitude has been reported as a reliable output of the retinal clock (33–37). This response reflects activity of On-center bipolar cells, and therefore cone photoreceptor signals from the outer retina. WT mice exhibit diurnal changes in cone-based ERG b-wave amplitude that are dependent on both CT and light history.

**Figure 4:**
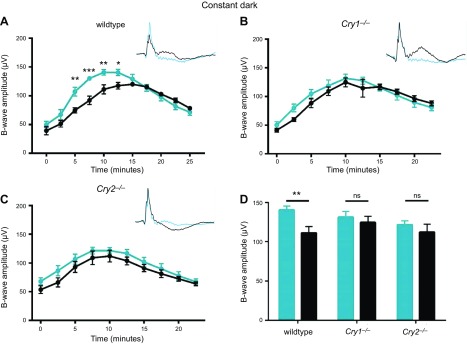
Photopic light-adapted ERG b-wave amplitudes are controlled in circadian manner and are dependent on both CRY1 and CRY2 expression. Data are presented as means ± sem. *A*) WT mice (*n* = 5) exhibit significantly larger photopic ERG b-wave amplitude at CT6 (blue) compared to CT18 (black). Retinal responses in mice were recorded over 25 min of light adaptation time. Representative traces (taken at 10 min of light adaptation) are shown in inset. *Post hoc* Bonferroni’s multiple comparisons tests show that WT mice have significantly higher b-wave amplitudes during subjective day (CT6) across range of 5 to 12.5 min of dark adaptation. *B*) *Cry1^−/−^* mice (*n* = 7) display no rhythm in photopic ERG b-wave amplitude. *Post hoc* Bonferroni’s multiple comparisons tests between all CT and light adaptation times were not significant. *C*) *Cry2^−/−^* mice (*n* = 7) demonstrate significant, but attenuated, difference in photopic ERG b-wave amplitudes at CT6 compared to CT18. Main effect of CT was found. However, *post hoc* Bonferroni’s multiple comparisons tests between all CT and light adaptation times were not significant. *D*) Summary of WT, *Cry1^−/−^*, and *Cry2^−/−^* data based on 10 min of light adaptation. WT mice demonstrate rhythm in photopic ERG b-wave amplitude between CT6 and CT18 at 10 min of light adaptation, while *Cry1^−/−^* and *Cry2^−/−^* mice do not. Comparable data were obtained under entrained conditions under LD cycles (Supplemental Fig. S2). *P < 0.05, **P < 0.01, ***P < 0.001, ****P < 0.0001.

Photopic light-adapted electroretinography was performed under constant darkness at CT6 and CT18 as in previous studies (33–37). ERG b-wave responses were recorded over 25 min of light adaptation time, and data were analyzed by 2-way ANOVA (light adaptation and CT). As expected, WT mice (*n* = 5) showed a significant main effect of light adaptation time (*F*_(10, 44)_ = 39.0, *P* < 0.0001), which peaked at around 10 min (**Fig. 4*A***). In addition, there was a significant effect of CT (*F*_(1, 44)_ = 28.8, *P* < 0.0001), and a significant interaction between CT × light adaptation time (*F*_(10, 44)_ = 5.01, *P* < 0.0001). *Post hoc* Bonferroni tests showed that WT mice have significantly higher b-wave amplitudes during the subjective day (CT6) across the range of 5 to 12.5 min of dark adaptation. In *Cry1^−/−^* mice (*n* = 7), significant differences of light adaptation time were still apparent (*F*_(9, 60)_ = 22.5, *P* < 0.0001), peaking at around 10 min, as in WT animals (Fig. 4*B*). However, there was no significant effect of CT (*F*_(1, 60)_ = 3.1, *P* = 0.083) and no significant interaction between CT × light adaptation time (*F*_(9, 60)_ = 1.13, *P* = 0.358). *Post hoc* Bonferroni tests showed no differences between CT at any duration of light adaptation. *Cry2^−/−^* mice (*n* = 7) showed a significant effect of light adaptation time (*F*_(9, 60)_ = 19.8, *P* < 0.0001), again peaking around 10 min (Fig. 4*C*). However, in these mice, there was a significant effect of CT (*F*_(1, 60)_ = 9.04, *P* = 0.0038) but no significant interaction between CT × light adaptation time (*F*_(9,60)_ = 0.167, *P* = 0.997). *Post hoc* Bonferroni tests showed no differences between CTs at any duration of light adaptation. As such, while a significant effect of CT was apparent, this circadian rhythm appears to be attenuated compared to WT animals.

A 10 min light adaptation protocol is most commonly used to study retinal rhythms, as this provides the maximal photopic b-wave amplitude (33, 37). When summarized in this manner, differences between the genotypes are immediately apparent, with both CRY1- and CRY2-deficient mice showing attenuated circadian rhythms (Fig. 4*D*). In addition, rhythms in ERG b-wave amplitude were also tested in all 3 genotypes under entrained conditions (12:12 LD cycle), comparing ZT6 and -18. Results were found to be comparable to those obtained under constant conditions (Supplemental Fig. S3).

### Rhythms in contrast sensitivity are affected in *Cry1^−/−^* but not *Cry2^−/−^* mice

Recent studies have demonstrated a circadian rhythm in visual contrast sensitivity that is also regulated by the retinal clock. The mechanism for this circadian phenomenon is through an interaction between dopamine D4 receptors, the clock gene *Npas2*, and adenylate cyclase 1. Because NPAS2 is only localized to the retinal ganglion cell layer, it appears that loss of the inner retinal clock alone is capable of abolishing rhythms in contrast sensitivity (28). As such, the circadian rhythm of contrast sensitivity appears to be primarily dependent on a circadian clock located within the inner retina. Conversely, circadian rhythms do not occur in visual acuity, suggesting that visual acuity and contrast sensitivity are independently regulated.

**Figure 5:**
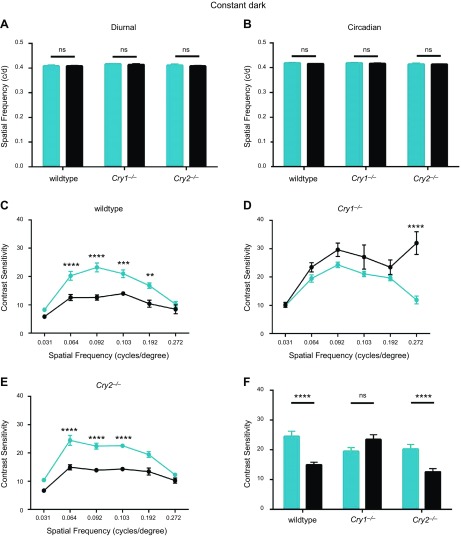
Circadian rhythm in contrast sensitivity is dependent on CRY1, but not CRY2. Responses were measured using the optokinetic nystagmus response and are shown as means ± sem. *A*) Visual acuity does not exhibit a diurnal variation, with no main effect of CT (*F*_(1, 42)_ = 0.2965, *P* = 0.5890), genotype (*F*_(2, 42)_ = 1.933, *P* = 0.1573) or interaction (*F*_(2,42)_ = 0.08302, *p* = 0.9205). *B*) No circadian variation was observed in visual acuity, with no main effect of CT (*F*_(1, 42)_ = 0.6298, *P* = 0.4319), genotype (*F*_(2, 42)_ = 1.267, *P* = 0.2923) or interaction (*F*_(2,42)_ = 0.1198, *P* = 0.8874). *C*) WT mice (*n* = 7) exhibit significantly higher contrast sensitivity at CT6 (blue) in contrast to CT18 (black). WT mice have significantly higher contrast sensitivities at spatial frequencies (cycles/degree) of 0.031, 0.064, 0.092, 0.103, and 0.192. *D*) *Cry1^−/−^* mice (*n* = 10) show no rhythms in contrast sensitivity at spatial frequencies that are rhythmic in WT mice. Interestingly, at the highest spatial frequency tested, *Cry1^−/−^* mice show significantly enhanced contrast sensitivity at CT18. *Cry1^−/−^* mice have significantly different contrast sensitivity at a spatial frequency of 0.272 but not at any other spatial frequencies tested. *E*) *Cry2^−/−^* mice (*n* = 10) show a circadian variation of contrast sensitivity similar to WT mice, suggesting CRY2 does not play a necessary role for this response. *Cry2^−/−^* mice have significantly higher contrast sensitivities at spatial frequencies (cycles/degree) of 0.064, 0.092, 0.103, and 0.192. *F*) Summary of contrast sensitivity of WT, *Cry1^−/−^* and *Cry2^−/−^* mice at a spatial frequency of 0.064 cycles/degree, corresponding to peak visual acuity. WT and *Cry2^−/−^* mice demonstrate a rhythm in contrast sensitivity between CT6 and CT18 at 0.064 cycles/degree spatial frequency, whilst *Cry1^−/−^* mice do not. *P < 0.05, **P < 0.01, ***P < 0.001, and ****P < 0.0001. Comparable data were obtained under entrained conditions under LD cycles (Supplemental Fig. S4).

Confirming previous data, we found that visual acuity did not show any diurnal or circadian variation between WT mice and mice lacking CRY1 or -2 (**Fig. 5*A, B***). Testing threshold spatial frequency, there was no significant effect of CT (*F*_(1, 42)_ = 0.630, *P* = 0.432) or genotype (*F*_(2, 42)_ = 1.27, *P* = 0.292), and no interaction between CT × genotype (*F*_(2,42)_ = 0.120, *P* = 0.887). We then tested the contrast sensitivity of mice at CT6 and CT18 (38), where circadian differences are known to exist (28). Contrast sensitivity was measured over a range of spatial frequencies ranging from 0.031 to 0.272 cycles/degree, and data were analyzed by 2-way ANOVA for spatial frequency and CT. As expected, in WT mice (*n* = 7), there was a significant effect of spatial frequency (*F*_(5, 54)_ = 39.0, *P* < 0.0001, Fig. 5*C*), with a peak around 0.064 to 0.092 cycles/degree, in agreement with published data (38). In addition, a significant effect of CT was apparent (*F*_(1, 54)_ = 150, *P* < 0.0001), with higher contrast sensitivity during the subjective day (CT6). A significant interaction occurred between CT × spatial frequency (*F*_(5, 54)_ = 5.44, *P* = 0.0004). *Post hoc* Bonferroni tests showed that WT mice have significantly higher contrast sensitivities at CT6 at spatial frequencies between 0.064 and 0.192 cycles/degree. In *Cry1^−/−^* mice (*n* = 10), the main effect of spatial frequency was still found to be significant (*F*_(5, 36)_ = 14.1, *P* < 0.0001, Fig. 5*D*), with a peak around 0.092 cycles/degree, but with a second peak at high spatial frequencies (0.272 cycles/degree). While there was a significant effect of CT (*F*_(1, 36)_ = 27.2, *P* < 0.0001) compared to WT animals, contrast sensitivity in *Cry1^−/−^* mice was higher during the subjective night (CT18). There was also a significant interaction between CT × spatial frequency (*F*_(5, 36)_ = 5.00, *P* = 0.0014). *Post hoc* Bonferroni tests show that *Cry1^−/−^* mice only show a significant difference in contrast sensitivity at a spatial frequency of 0.272, but not at any other spatial frequencies tested. As such, the main effect of CT appears to be due to the significant difference at 0.272 cycles/degree (high acuity).

*Cry2^−/−^* mice (*n* = 10) again showed a significant effect of spatial frequency (*F*_(5, 36)_ = 26.9, *P* < 0.0001), peaking at 0.064 cycles/degree (Fig. 5*E*). There was also a significant effect of CT (*F*_(1, 36)_ = 91.2, *P* < 0.0001), with higher contrast sensitivity during the subjective day (CT6), comparable with WT animals. There was a significant interaction between CT × spatial frequency (*F*_(5, 36)_ = 4.73, *P* = 0.002). *Post hoc* Bonferroni tests showed that *Cry2^−/−^* mice have significantly higher contrast sensitivities at spatial frequencies between 0.031 and 0.103 cycles/degree, comparable with WT animals.

When contrast sensitivity is compared at the peak of visual acuity, 0.064 cycles/degree as reported previously (38), significant circadian rhythms are clearly apparent in WT and CRY2-deficient mice, but not in CRY1-deficient animals (Fig. 5*F*). Rhythms in contrast sensitivity were also tested in all 3 genotypes under entrained conditions, comparing ZT6 and -18. Results were found to be comparable to those obtained under constant conditions (Supplemental Fig. S4).

### Rhythms in pupillary responses are attenuated in *Cry1^−/−^* but not *Cry2^−/−^* mice

**Figure 6:**
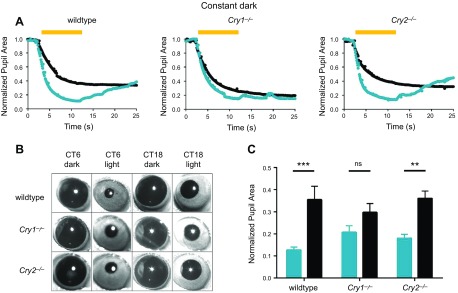
PLR is controlled in a circadian manner and is dependent on CRY1 but not CRY2. *A*) Representative sample kinetics of PLR at CT6 (blue) and CT18 (black) for WT, *Cry1^−/−^*, and *Cry2^−/−^* mice. Yellow bar indicates stimulus duration at 2 to 12 s of recording. WT and *Cry2^−/−^* mice demonstrated attenuated PLR at CT18 compared to CT6. However, *Cry1^−/−^* mice have similar PLR at CT6 and -18. *B*) Set of pupil images demonstrating maximum constriction of pupils at CT6 and CT18 for WT, *Cry1^−/−^*, and *Cry2^−/−^* mice. *C*) Summary histogram for WT (*n* = 5), *Cry1^−/−^* (*n* = 7), and *Cry2^−/−^* (*n* = 7) mouse responses at CT6 (blue) and CT18 (black). Data are presented as means ± sem. Maximum pupil constriction is significantly different between CT6 and CT18 in WT mice and *Cry2^−/−^* mice, but not in *Cry1^−/−^* mice. Comparable data were obtained under entrained conditions under LD cycles (Supplemental Fig. S4).

The pupillary light response (PLR) exhibits both circadian and diurnal rhythms in response magnitude (21). We therefore compared circadian rhythms in the PLR in WT (*n* = 5), *Cry1^−/−^* (*n* = 7), and *Cry2^−/−^* (*n* = 7) mice (**Fig. 6*A–C***). Comparing circadian rhythms in maximum constriction, pupillary responses were found to be significantly attenuated at CT18 in WT mice (Student’s *t* test, *P* = 0.00022). While no significant circadian difference was detected in *Cry1^−/−^* mice (Student’s *t* test, *P* = 0.058), *Cry2^−/−^* mice showed significant circadian differences, comparable to WT animals (Student’s *t* test, *P* = 0.001).

In summary, circadian rhythms in the PLR were detected in WT and CRY2-deficient mice but were abolished in mice lacking CRY1. Rhythms in the PLR were also tested in all 3 genotypes under entrained conditions, comparing ZT6 and -18. Interestingly, *Cry1^−/−^* mice showed a PLR rhythm under ZT conditions, which is likely due to light masking, similar to *Cry1^−/−^;Cry2^−/−^* mice, which show behavioral activity masking under LD conditions (Figs. 1–6). Results were found to be comparable to those obtained under constant conditions (Supplemental Fig. S5). Finally, to determine whether changes in melanopsin expression could account for differences in circadian rhythms in PLR, we compared melanopsin expression in *Cry1^−/−^* and *Cry2^−/−^* mice *vs.* WT controls. No differences in levels of melanopsin expression, or numbers, anatomy, or distribution of ipRGCs were evident in either CRY-deficient mouse line (Supplemental Fig. S6).

## DISCUSSION

Here we show not only that CRY1 is widely expressed in cones, amacrine cells, and retinal ganglion cells of the mammalian retina but also that it plays an essential role in the generation of functional retinal rhythms. Mice lacking CRY1 show an abolition or attenuation of all aspects of retinal circadian physiology investigated, including PER2::LUC rhythms, photopic ERG b-wave amplitude, contrast sensitivity, and PLR. In contrast, CRY2 localization in the retina was challenging, and we were unable to find a reliable CRY2 antibody. Compared to CRY1, loss of CRY2 results in an attenuation of rhythms in ERG b-wave amplitude but no effect on retinal rhythms of PER2::LUC bioluminescence, contrast sensitivity, or PLR. Collectively, these data suggest that CRY1 is the preeminent CRY involved in retinal rhythm generation. Our data suggest that both CRY1 and -2 may be necessary for the generation of outer retinal rhythms, reflected by rhythms in photopic ERG b-wave amplitude. In contrast, CRY1 but not CRY2 plays a key role in the inner retinal clock, reflected by rhythms in PER2::LUC bioluminescence, contrast sensitivity, and pupil constriction.

Surprisingly, previous immunohistochemical studies have detected CRY2 but failed to detect CRY1 protein expression in the mouse retina (9, 11). These studies have used commercially available antibodies without knockout tissue controls. Therefore, it is possible that the expression of CRYs previously described in the mouse retina may not be specific (Supplemental Fig. S1). We have validated both antisera used in this study, using both heterologous expression in cell lines (Supplemental Fig. S2) and knockout controls (Fig. 1 and Supplemental Fig. S1). Moreover, our findings agree with numerous studies that have reported expression of both *Cry1* and *-2* mRNA in the mammalian retina, including studies using *in situ* hybridization that report more widespread expression (15, 39–41). It is unclear why CRY2 expression was so difficult to reliably localize in the retina when these antisera have been successfully used in other tissues.

CRY1 and -2 have frequently been assumed to play interchangeable roles within the TTFL. Our results indicate that at the level of the retina, this is an oversimplification. This is consistent with emerging data where CRY1 and -2 appear to serve distinct and separate functions (24, 42, 43), including differences in the timing of their genomic interactions (44). It has previously been suggested that the mechanism underlying circadian rhythm generation in the retina differs from that in the SCN. Studies of retinal PER2::LUC rhythms have shown that the loss of *Per1*, *Cry1*, or *Arntl* (*Bmal1*) is sufficient to induce arrhythmicity, while SCN rhythms are maintained in the absence of *Per1* or *Cry1*, albeit with a change in period (43). As in the SCN, we find that loss of CRY1 results in a shorter circadian period in the retina, whereas loss of CRY2 results in a longer retinal circadian period. However, unlike the SCN, we show that retinal rhythm stability is dependent on CRY1 but not CRY2. Together, these findings suggest that the retinal clock may differ from the master clock in the SCN. This may be due to differences in how retinal circadian clocks are synchronized compared to the central SCN clock. Cell–cell communication involving vasoactive intestinal polypeptide (VIP) and arginine vasopressin (AVP) play a critical role in the SCN neuronal network (45, 46). In contrast, the more diffuse neuronal network of the retinal clock appears to depend upon dopamine and melatonin (3), as well as coupling *via* gap junctions (47).

Data from rhythms in retinal physiology in *Cry1^−/−^* mice recorded in the subjective night (CT18) show comparable responses to those observed in the subjective day (CT6). While loss of retinal rhythms may be expected to result in an averaging of daytime and nighttime responses, *Cry1^−/−^* mice appear to display daytime responses at all phases. Similar findings have been reported in *Cry1^−/−^;Cry2^−/−^* mice (35), where these arrhythmic animals demonstrate daylike photopic ERG b-wave responses at both ZT6 and -18. In contrast, retina-specific *Bmal1^−/−^* mice demonstrate attenuated nightlike responses at ZT6 and -18 (37). This difference has been attributed to the contrasting roles of *Bmal1* and *Cry1* in the TTFL. As a transcriptional activator, BMAL1 regulates the positive limb of the TTFL, regulating clock genes and clock-controlled genes *via* its activity at E-box enhancers. We suggest that processes under control of the E box may be necessary for the increased sensitivity of the retina during the day. Conversely, as CRY1 forms part of the negative arm of the TTFL, inhibiting the positive drive of CLOCK and BMAL1, this will result in no deactivation of the E-box–driven increase in sensitivity. As a result, the antiphase activity of BMAL1 and CRY1 may account for the different phasing of retinal clocks in their absence. Further evidence comes from studies on NPAS2, a paralog of CLOCK, the binding partner of BMAL1. Consistent with the differing retinal effects of positive and negative components of the TTFL, *Npas2^−/−^* mice demonstrate nightlike contrast sensitivity responses at both CT6 and -18. The contrast sensitivity data that we present herein provides further support for this model, where *Cry1^−/−^* mice demonstrate a daylike response at both CT6 and -18. To our knowledge, circadian rhythms in photopic ERGs or contrast sensitivity have not been investigated in other clock gene knockouts. However, mice lacking *Per1* and *-2* show attenuated daytime PLR responses (21), which may correspond to the reduced sensitivity PLR responses observed at night. Contrary to the model proposed above, mice lacking *Bmal1* also show reduced PLR sensitivity (21). This suggests that additional mechanisms may be involved in regulating the PLR. In contrast, mice lacking *Rev-erbα* (NR1D1) show enhanced sensitivity to light in ERG and PLR responses, consistent with the role of *Rev-erbα* in providing negative feedback on BMAL1 and E-box activation. However, these data are additionally complicated by a role of *Rev-erbα* in regulating melanopsin expression (48).

While the PLR has played a critical role in characterizing the non–image-forming responses to light, evidence for diurnal and circadian rhythms has only recently been described (21). The PLR demonstrates clear circadian rhythms in sensitivity, with reduced responses during the subjective night. This circadian rhythm in sensitivity is attenuated in CRY1-deficient mice but retained in CRY2-deficient animals, again consistent with a preeminent role of CRY1 in retinal rhythms. It is currently unclear whether rhythms in PLR sensitivity are driven by the retinal clock or by rhythms in the central neural pathways mediating this response, including the autonomic nervous system.

Surprisingly, we found a significant but aberrant circadian variation in contrast sensitivity at high spatial frequencies in *Cry1^−/−^* mice, with higher contrast sensitivity during the subjective night (CT18) at 0.272 cycles/degree. Not only is this response the reverse of what is seen in WT and *Cry2^−/−^* animals but it also occurs consistently under both entrained (ZT) and constant (CT) conditions, and only at the highest spatial frequencies (Fig. 5*D* and Supplemental Fig. S4*B*). No previous studies of circadian retinal physiology have reported such changes (28, 35, 37). This suggests that while circadian rhythms in contrast sensitivity are attenuated at most spatial frequencies, there may be some residual, although aberrant, circadian function in *Cry1^−/−^* mice. Potential explanations for this finding are that there may be a cellular subpopulation in which CRY2 is capable of partially substituting for the loss of CRY1, or that functional extraretinal clocks may be influencing contrast sensitivity at these spatial frequencies. Given the high spatial frequencies involved and the limited overlap of CRY1 and -2 expression, this may reflect changes in cone function.

Our data also demonstrate that CRY1-deficient mice provide a valuable model to study the role of the retinal clock because, unlike *Cry1/2^−/−^* mice, these animals still possess a central circadian clock (although one with a shorter circadian period). It is unclear whether the different retinal phenotypes observed in clock gene knockouts may relate to their role in the regulation of tissue-specific transcriptional profiles. Because CLOCK and BMAL1 are transcription factors that drive the tissue-specific expression of numerous other target genes, loss of these critical factors may produce effects independent of their role in the circadian clock (49–53). As such, while conditional targeting of retinal BMAL1 provides an ideal way of specifically ablating retinal rhythms (37), this may result in noncircadian changes in retinal function. In contrast, CRY1-deficient mice provide a model in which to study the role of retinal circadian rhythms in which the positive limb of the circadian clock is preserved but the circadian rhythm of transcriptional repression is lost.

In summary, our data show that circadian rhythms in retinal physiology depend primarily on CRY1, while CRY2 appears largely redundant. Using multiple assays of retinal circadian rhythms, we show that *Cry1^−/−^* mice show attenuated or abolished retinal rhythms, whereas *Cry2^−/−^* animals only show deficits in ERG responses. These retinal functions can be correlated with the expression pattern of CRY1 and -2 in the mouse retina, with CRY1 expression detected in multiple cell types, whereas CRY2 expression is more limited. These findings suggest that CRY1 and -2 play distinct and separate roles in the regulation of retinal circadian physiology.

## Supplementary Material

This article includes supplemental data. Please visit *http://www.fasebj.org* to obtain this information.

## Abbreviations

CRY: cryptochrome
CT: circadian time
ERG: electroretinogram
eYFP: enhanced yellow fluorescent protein
ipRGC: intrinsically photosensitive retinal ganglion cell
LD: light/dark
PER: period
PER2::LUC: period 2::luciferase
PLR: pupillary light response
SCN: suprachiasmatic nuclei
TTFL: transcriptional–translational feedback loop
UVS: ultraviolet sensitive
WT: wild type
ZT: zeitgeber time
