# Supplementary material for: Differential roles for cryptochromes in the mammalian retinal clock
Source: FASEB J. 2018 Mar 21;32(8):4302–14. doi: 10.1096/fj.201701165RR (PMC6071063; doi:10.1096/fj.201701165RR)

SUPPLEMENTARY DATA

Figure S1. CRY2 expression in the mouse retina. (A) CRY2 was examined using the Alpha Diagnostics (ADI) commercial CRY2 antibody. ADI CRY2 antibody (green) provides a signal in multiple layers of the wildtype mouse retina. (B) Comparable immunoreactivity was obtained in $Cry2^{-/-}$ tissue, suggesting that this signal was not specific. (C) Alternative CRY2 antisera (green) were subsequently used. No reliable signal was obtained in the mouse retina. (D) Again, comparable signal for CRY2 (green) was observed in $Cry2^{-/-}$ retina, with slightly lower signal in the photoreceptor layer. DAPI staining (blue) in cell nuclei is also shown. Merge indicates all colour channels (green and blue) combined. Scale bar = 20µm.

Figure S2. CRY1 and CRY2 antibodies are validated and confirmed to recognize CRY1 and CRY2, respectively, without non-specific reactivity in HEK293 cells transfected with CRY1 and CRY2. Scale bar indicates 10um. (A) Cry1-HA transfected cells are recognized by CRY1 antibody (left) and HA antibody control (right). (B) Cry2-HA transfected cells are not recognized by CRY1 antibody (left), but are recognized by HA antibody control (right). (C) Cry2-HA transfected cells are recognized by CRY2 antibody (left) and HA antibody control (right). (D) Cry1-HA transfected cells are not recognized by CRY2 antibody (left), but are recognized by HA antibody control (right).

Figure S3. Photopic light-adapted electroretinogram b-wave amplitudes are also controlled in a diurnal manner under entrained conditions and are dependent on both Cry1 and Cry2 expression. Data are represented as means ± SEM. (A) Wildtype mice (n=5) exhibit a significantly larger photopic electroretinogram b-wave amplitude at ZT6 (blue) compared to ZT18 (black). Retinal responses in mice were recorded over 25 minutes of light adaptation time. Representative traces (taken at 10 minutes of light adaptation) are shown inset. Post-hoc Bonferroni's multiple comparisons test show that wildtype mice have significantly higher b-wave amplitudes during the day (ZT6) across the range of 5-12.5 minutes of dark adaptation. (B) $Cry1^{-/-}$ mice (n=7) display no rhythm in photopic ERG b-wave amplitude. Post-hoc Bonferroni's multiple comparisons test between all zeitgeber time and light adaptation times were not significant. (C) $Cry2^{-/-}$ mice (n=7) demonstrate a significant difference in photopic ERG b-wave amplitudes at ZT6 compared to ZT18. Post-hoc Bonferroni's multiple comparisons test show that $Cry2^{-/-}$ mice have significantly higher b-wave amplitudes at light adaptation times of 5 and 7.5 minutes of duration. (D) Summary of wildtype, $Cry1^{-/-}$ and $Cry2^{-}$

$^{/-}$ data based on 10 minutes of light adaptation are shown. Wildtype and Cry2$^{-/-}$ mice demonstrate a rhythm in photopic ERG b-wave amplitude between ZT6 and ZT18 at 10 minutes of light adaptation, whilst Cry1$^{-/-}$ mice do not. *indicates $p<0.05$, **indicates $p<0.01$, ***indicates $p<0.001$, and ****indicates $p<0.0001$.

Figure S4. Under entrained conditions diurnal rhythms in contrast sensitivity are also dependent on Cry1, but not Cry2. Data are represented as means ± SEM. (A) Wildtype mice (n=7) exhibit significantly higher contrast sensitivity at ZT6 (blue) in contrast to ZT18 (black), as measured using the optokinetic nystagmus response. Wildtype mice have significantly higher contrast sensitivities at spatial frequencies (cycles/degree) of 0.064, 0.092 and 0.103. (B) Cry1-/- mice (n=10) show no rhythms in contrast sensitivity at spatial frequencies that are rhythmic in wildtype mice. Interestingly, at the two highest spatial frequencies tested, Cry1-/- mice show significantly enhanced contrast sensitivity at ZT18. Cry1-/- mice have significantly different contrast sensitivity at spatial frequencies of 0.192 or 0.272 but not at any other spatial frequencies tested. (C) Cry2-/- mice (n=10) show a diurnal variation of contrast sensitivity similar to wildtype mice, suggesting Cry2 does not play a necessary role for this response. Cry2-/- mice have significantly higher contrast sensitivities at the same spatial frequencies (cycles/degree) as wildtype mice, at 0.064, 0.092 and 0.103 cycles/degree. (D) Summary of contrast sensitivity of wildtype, Cry1-/- and Cry2-/- mice at a spatial frequency of 0.064 cycles/degree, corresponding to peak visual acuity. Wildtype and Cry2-/- mice demonstrate a rhythm in contrast sensitivity between ZT6 and ZT18 at 0.064 cycles/degree spatial frequency, whilst Cry1-/- mice do not. *indicates $p<0.05$, **indicates $p<0.01$, ***indicates $p<0.001$, and ****indicates $p<0.0001$.

Figure S5. The pupillary light response also exhibits a diurnal rhythm under entrained conditions. (A) Representative sample kinetics of pupillary light responses at ZT6 (blue) and ZT18 (black) for wildtype, Cry1$^{-/-}$, and Cry2$^{-/-}$ mice. The yellow bar indicates stimulus duration at 2s-12s of recording. All mice demonstrated significantly attenuated pupillary light responses at ZT18 compared to ZT6. (B) Set of pupil pictures demonstrating that the maximum constriction of pupils at ZT6 and ZT18 for wildtype, Cry1$^{-/-}$, and Cry2$^{-/-}$ mice. (C) Summary

histogram for wildtype (n=5), Cry1$^{-/-}$ (n=7) and Cry2$^{-/-}$ (n=7) mouse responses at ZT6 (blue) and ZT18 (black). Data are represented as means ± SEM. Maximum pupil constriction is significantly different between ZT6 and ZT18 in wildtype mice ($p = 0.017$), in Cry1$^{-/-}$ mice ($p = 0.00023$), and in Cry2$^{-/-}$ mice ($p = 0.00077$).

Figure S6. Representative images of melanopsin-expressing retinal ganglion cells in wildtype (WT), Cry1-deficient (Cry1$^{-/-}$) and CRY2-deficient (Cry2$^{-/-}$) mouse retina. No differences in melanopsin expression were evident.

Figure S1

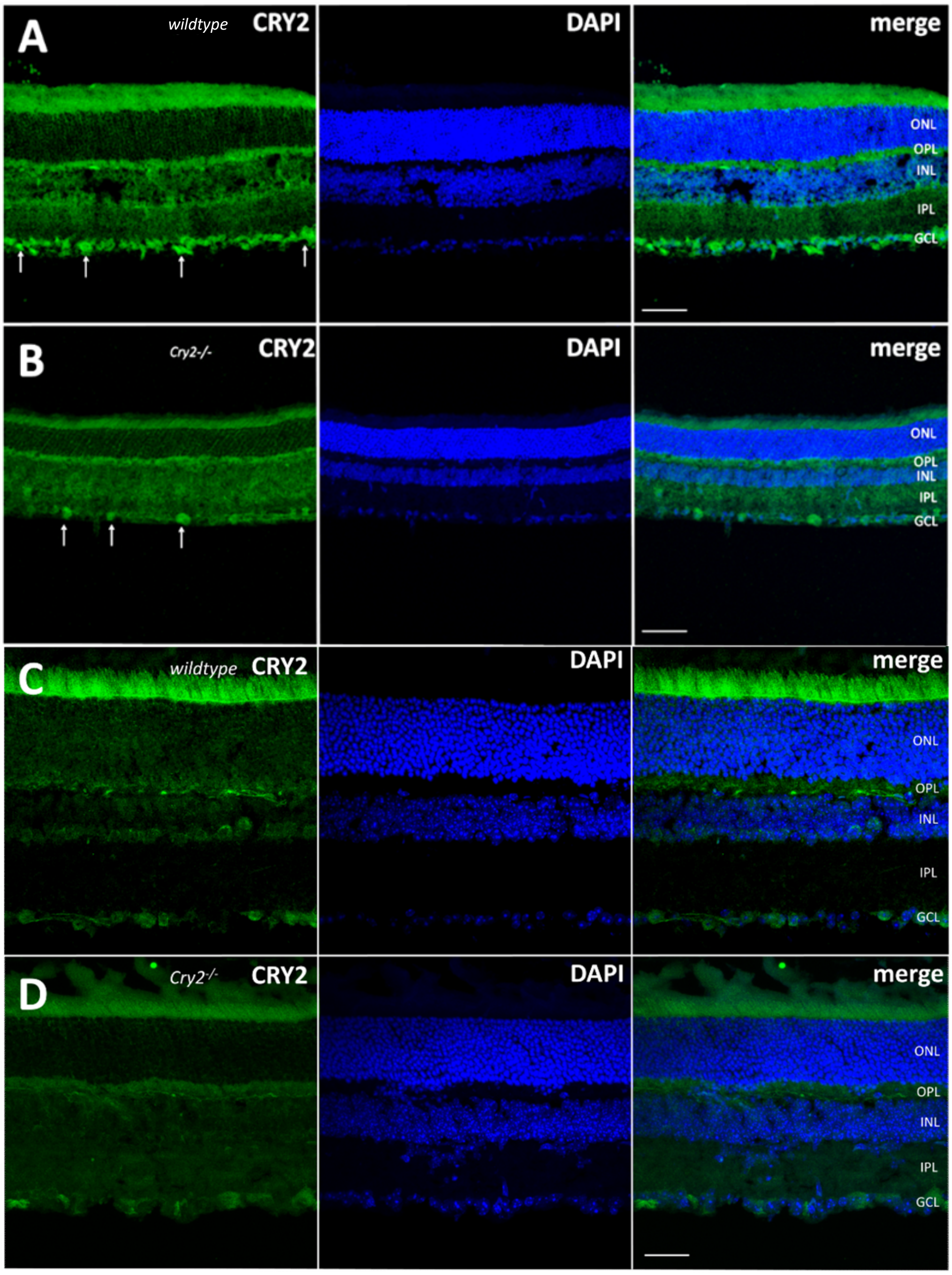

A
wildtype
CRY2
DAPI
merge
ONL
OPL
INL
IPL
GCL
B
Cry2-/-
CRY2
DAPI
merge
ONL
OPL
INL
IPL
GCL
C
wildtype
CRY2
DAPI
merge
ONL
OPL
INL
IPL
GCL
D
Cry2-/-
CRY2
DAPI
merge
ONL
OPL
INL
IPL
GCL

Figure S2

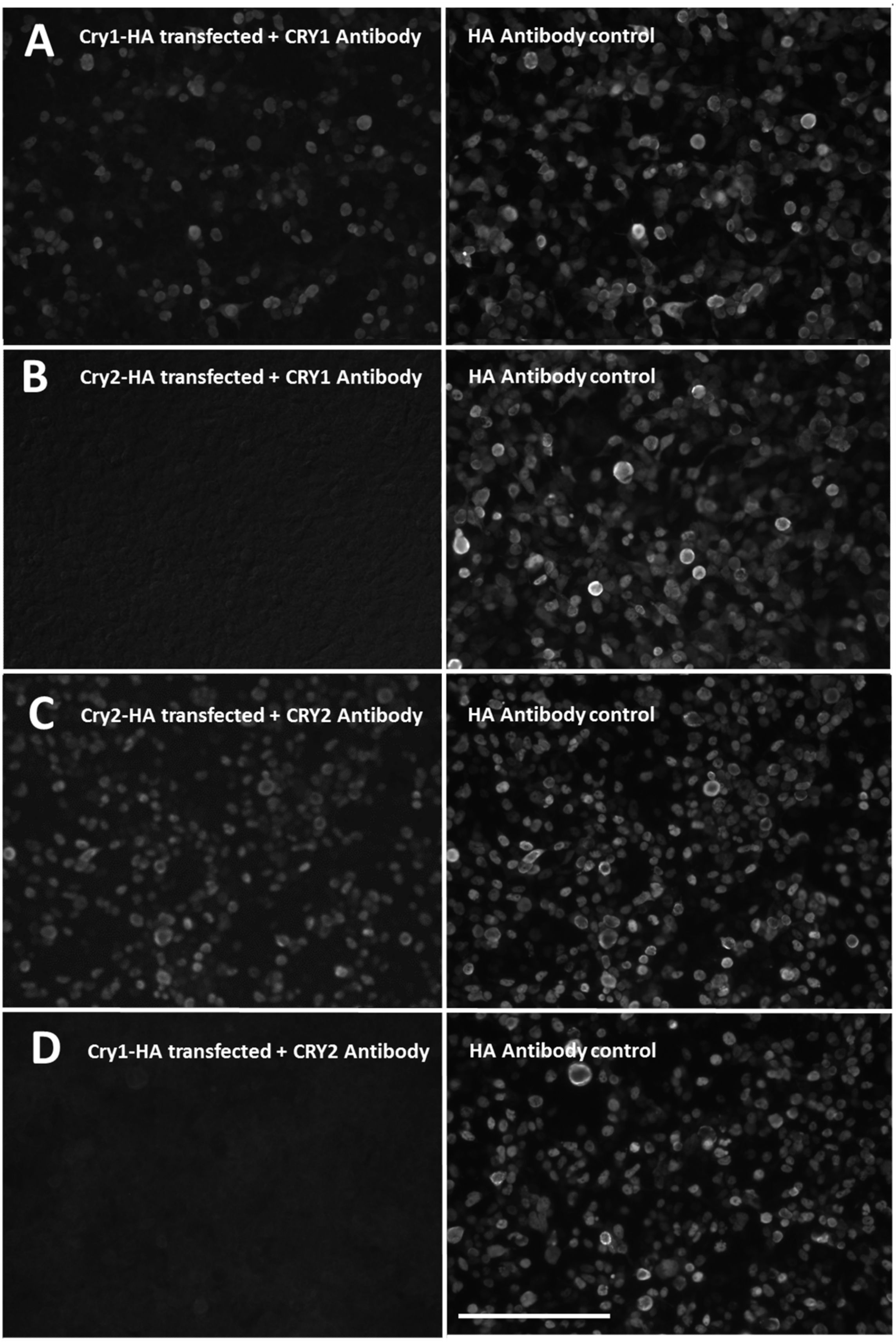
A
Cry1-HA transfected + CRY1 Antibody
HA Antibody control
B
Cry2-HA transfected + CRY1 Antibody
HA Antibody control
C
Cry2-HA transfected + CRY2 Antibody
HA Antibody control
D
Cry1-HA transfected + CRY2 Antibody
HA Antibody control

Figure S3

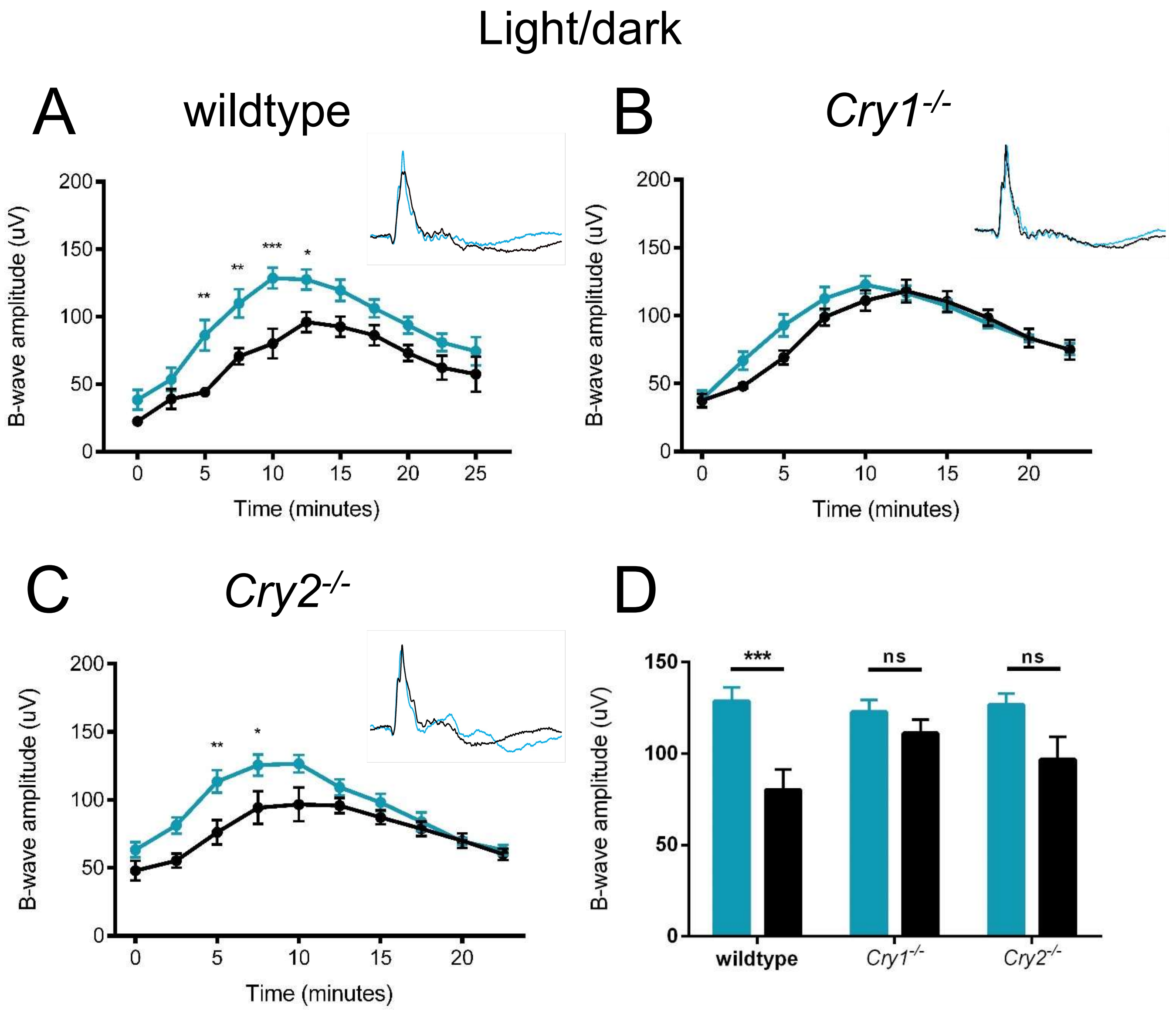
Light/dark
A
wildtype
200
150
100
50
0
B-wave amplitude (uV)
0
5
10
15
20
25
Time (minutes)
**
**
***
*
B
Cry1-/-
200
150
100
50
0
B-wave amplitude (uV)
0
5
10
15
20
Time (minutes)
C
Cry2-/-
200
150
100
50
0
B-wave amplitude (uV)
0
5
10
15
20
Time (minutes)
**
*
D
150
100
50
0
B-wave amplitude (uV)
***
ns
ns
wildtype
Cry1-/-
Cry2-/-

Figure S4

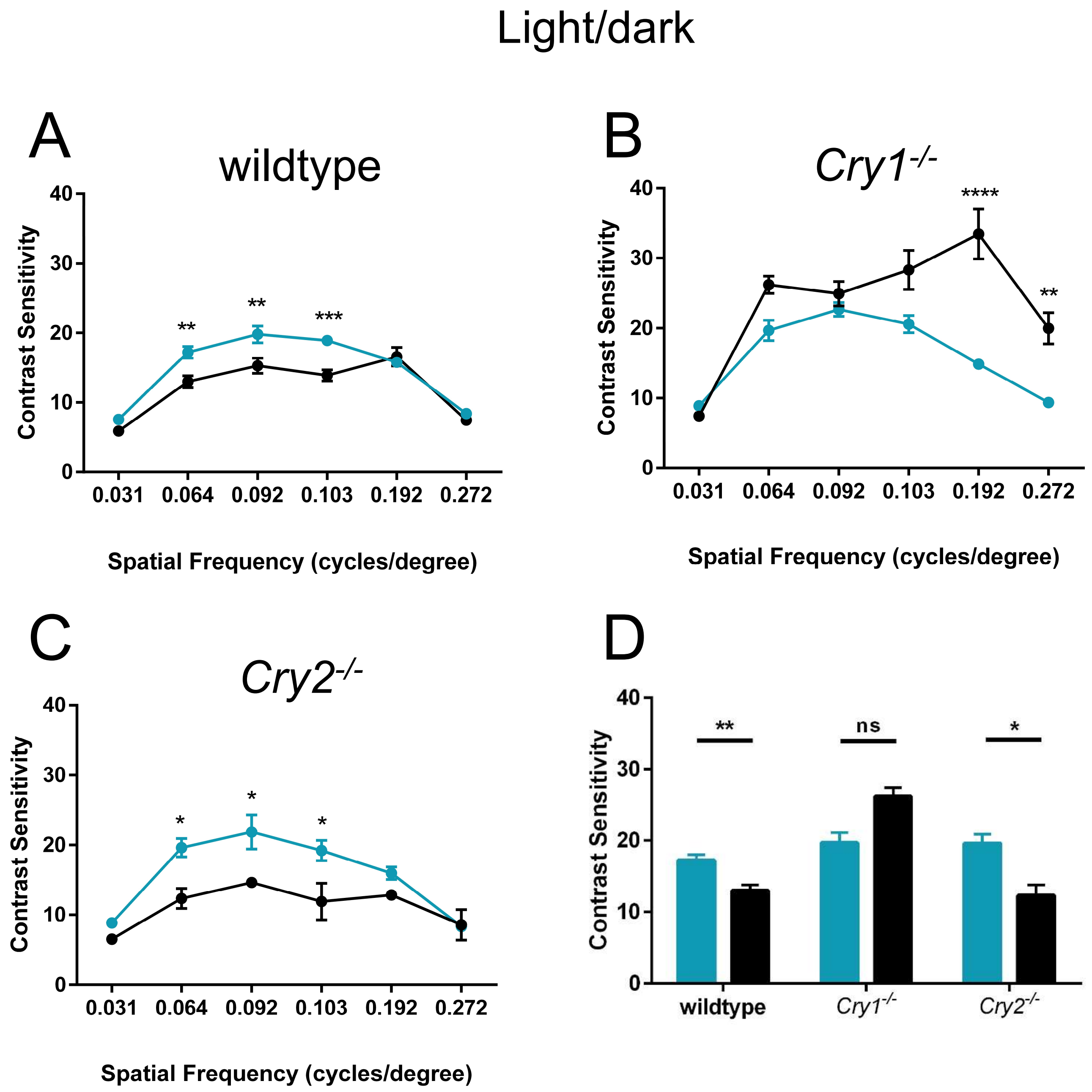

Light/dark
A
wildtype
Contrast Sensitivity
40
30
20
10
0
**
**
***
0.031 0.064 0.092 0.103 0.192 0.272
Spatial Frequency (cycles/degree)
B
Cry1-/-
Contrast Sensitivity
****
**
0.031 0.064 0.092 0.103 0.192 0.272
Spatial Frequency (cycles/degree)
C
Cry2-/-
Contrast Sensitivity
*
*
*
0.031 0.064 0.092 0.103 0.192 0.272
Spatial Frequency (cycles/degree)
D
Contrast Sensitivity
**
ns
*
wildtype
Cry1-/-
Cry2-/-

Figure S5

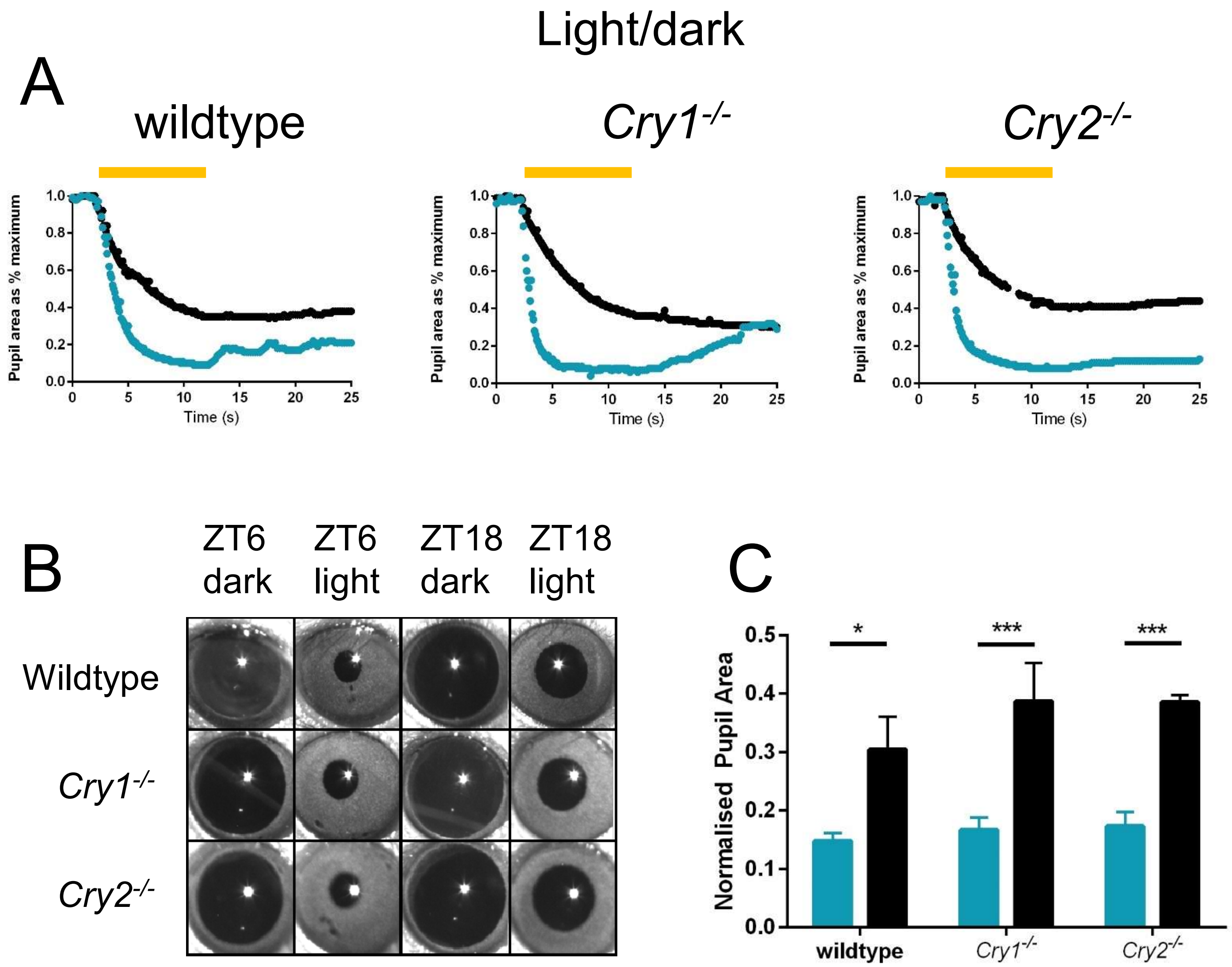
Light/dark
A
wildtype
Cry1-/-
Cry2-/-
Pupil area as % maximum
Time (s)
B
ZT6 dark
ZT6 light
ZT18 dark
ZT18 light
Wildtype
Cry1-/-
Cry2-/-
C
Normalised Pupil Area
*
***
***
wildtype
Cry1-/-
Cry2-/-

Figure S6

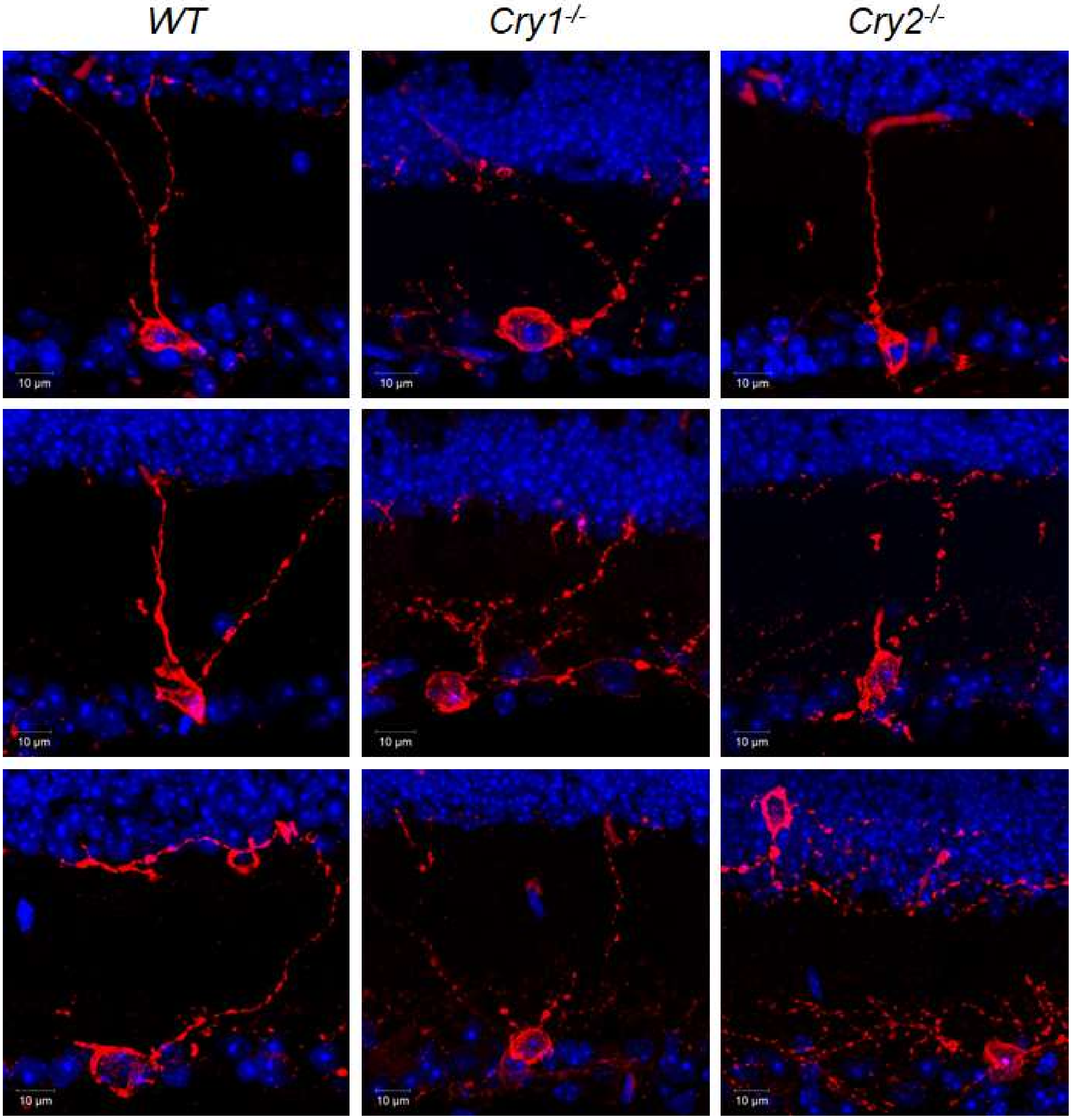

Supplement: Supplementary file 1 [file fj.201701165RR.sf1.pdf]
